# Ketogenic diet induces skeletal muscle atrophy via reducing muscle protein synthesis and possibly activating proteolysis in mice

**DOI:** 10.1038/s41598-019-56166-8

**Published:** 2019-12-23

**Authors:** Reiko Nakao, Tomoki Abe, Saori Yamamoto, Katsutaka Oishi

**Affiliations:** 1 Biological Clock Research Group, Biomedical Research Institute, National Institute of Advanced Industrial Science and Technology (AIST), Tsukuba, Ibaraki 305-8566 Japan; 2 Department of Applied Biological Science, Graduate School of Science and Technology, Tokyo University of Science, Noda, Chiba 278-8510 Japan; 3 Department of Computational and Medical Sciences, Graduate School of Frontier Sciences, The University of Tokyo, Kashiwa, Chiba 277-0882 Japan; 4 School of Integrative and Global Majors (SIGMA), University of Tsukuba, Tsukuba, Ibaraki 305-8577 Japan

**Keywords:** Homeostasis, Metabolic diseases

## Abstract

Ketogenic diets (KD) that are very high in fat and low in carbohydrates are thought to simulate the metabolic effects of starvation. We fed mice with a KD for seven days to assess the underlying mechanisms of muscle wasting induced by chronic starvation. This diet decreased the weight of the gastrocnemius (Ga), tibialis anterior (TA) and soleus (Sol) muscles by 23%, 11% and 16%, respectively. The size of Ga, TA, Sol muscle fibers and the grip strength of four limbs also significantly declined by 20%, 28%, 16% and 22%, respectively. The muscle atrophy-related genes *Mafbx*, *Murf1*, *Foxo3*, *Lc3b* and *Klf15* were upregulated in the skeletal muscles of mice fed with the KD. In accordance with the reduced expression of anabolic genes such as *Igf1*, surface sensing of translation (SUnSET) analyses of fast-twitch Ga, TA and Sol muscles revealed that the KD suppressed muscle protein synthesis. The mRNA expression of oxidative stress-responsive genes such as *Sod1* was significantly increased in all muscles examined. In addition to hypercorticosteronemia, hypoinsulinemia and reduced IGF-1, oxidative stress might also be involved in KD-induced muscle atrophy. Feeding mice with a KD is a novel experimental animal model of muscle-wasting induced by chronic starvation.

## Introduction

Skeletal muscle comprises 40% of the body mass and it functions in locomotion, postural support, glucose uptake and fatty acid oxidation. Skeletal muscle consists of distinct fast-twitch glycolytic and slow-twitch oxidative fibers. Balanced muscle protein synthesis and degradation can retain skeletal muscle mass and muscle performance. Muscle protein metabolism varies according to nutritional and environmental circumstances. It increases in response to food intake, exercise and anabolic hormones such as insulin and insulin-like growth factor 1 (IGF-1), and decreases in response to starvation, aging, muscle disuse, loss of neural input, cancer and catabolic hormones such as glucocorticoid (GC)^1^.

The physiological and molecular events that occur in rodents deprived of food for 24–48 h have been determined in a study of the mechanism of muscle mass loss^2^. Total food deprivation suppresses insulin secretion and increases GC production, then activates muscle protein catabolism to release alanine and other amino acids as a source of hepatic gluconeogenesis. Increased fatty acid oxidation leads to the production of ketone bodies. Depriving rodents of food also induces hyperactivity. Impaired insulin signaling and an increase in endogenous GC could trigger the degradation of skeletal muscle proteins by activating the ubiquitin-proteasome (UPS) and autophagy systems^3^. The fast-twitch fibers in the gastrocnemius (Ga) and tibialis anterior (TA) muscles are more sensitive to atrophy induced by fasting than slow-twitch fibers^3^ possibly because they are prone to GC-induced muscle atrophy^4,5^. The stimulation of these proteolytic systems is mediated through increased expression of the muscle-specific ubiquitin ligases, muscle atrophy F-box (MAFbx) and muscle RING finger 1 (MuRF1)^6–10^, as well as autophagy^11^. Activation of these muscle atrophy-related genes is regulated by GC-regulated transcription factors such as glucocorticoid receptor (GR)^8,10,12^, FOXOs^11^ and KLF15^13^. However, these molecular events might be transient in rodents that have fasted for 24–48 h and might not accurately reflect the effects of chronically poor nutrition. In fact, the amount of total muscle proteolysis varies daily in humans deprived of food^3^. Furthermore, Lange *et al*. sequentially analyzed skeletal muscle in rats after a 48-h fast and identified a rapid, transient starvation signal. The intramuscular AMP/ATP ratio, an indicator of cellular energy status, significantly increased from ~1.5- to 12.5-fold between 6 and 24 h, respectively, then started to decrease at 48 h but remained ~5-fold higher than that of non-fasted mice^14^. The phosphorylation of AMP-activated protein kinase (AMPK) also transiently increased at 6 and 12 h, then returned to baseline at 48 h. These results suggest that intracellular energy status remarkably varies after a short fast. We consider that muscle-wasting is caused by chronic and mild malnutrition rather than fasting in humans. Thus, experimental food withdrawal is limited as an animal model of the effects of chronically poor nutritional status on skeletal muscle.

Ketogenic diets (KD) comprising high-fat, low-carbohydrate and protein contents have been included in weight loss strategies for both obese and non-obese individuals. Such diets mimic the metabolic status of fasting or caloric restriction and are based on theoretical concepts of the effects of dietary component ratios on energy expenditure^15^. The present study aimed to determine degrees of muscle atrophy in mice fed with a KD.

## Results

### Ketogenic diet mimics chronic starvation

**Figure 1:**
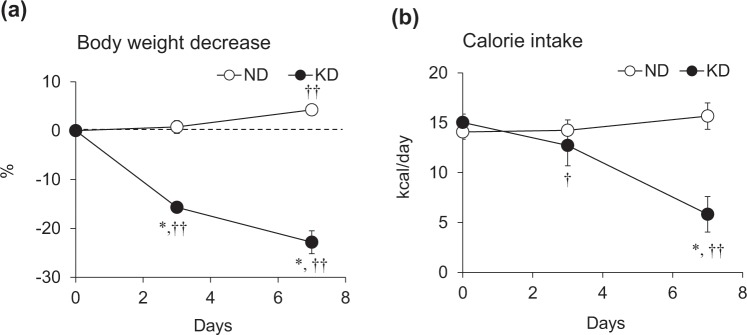
Body weight and caloric intake during ketogenic diet consumption. Rates of body weight decreases (**a**) and calorie intake (**b**) in mice fed with normal (ND; unfilled circles) or ketogenic (KD; filled circles) diets for 0, 3 and 7 days. Results are shown as means ± SEM (n = 7–8 per group). ^*^P < 0.01 (ND vs. KD). ^†^*P* < 0.05 and ^††^*P* < 0.01 (Day 0 vs. Days 3 and 7).

We initially measured the body weight (BW) and caloric intake of mice fed with a KD or the AIN93M normal diet (ND). The BW of mice fed with the ND increased by 4.3% over the experimental period (Fig. 1a), but rapidly decreased by 16% in mice given the KD during the first three days, then by 9% during the next four days (Fig. 1a). Caloric intake did not significantly differ between ND and KD mice on day 3 but decreased by 65% on day 7 in those fed with the KD (Fig. 1b).

**Figure 2:**
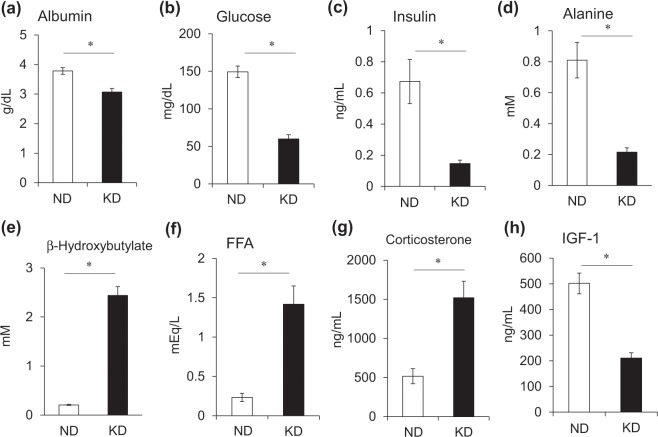
Plasma metabolic parameters. Plasma concentrations of albumin (**a**), glucose (**b**), insulin (**c**), alanine (**d**), β-hydroxybutyrate (**e**), free fatty acids (FFA) (**f**), corticosterone (**g**), and insulin-like growth factor 1 (IGF-1) (**h**) in mice fed with normal (ND; unfilled squares) or ketogenic (KD; filled squares) diet for seven days. Results are shown as means ± SEM (n = 7–8 per group). ^*^P < 0.01 (ND vs. KD).

**Figure 3:**
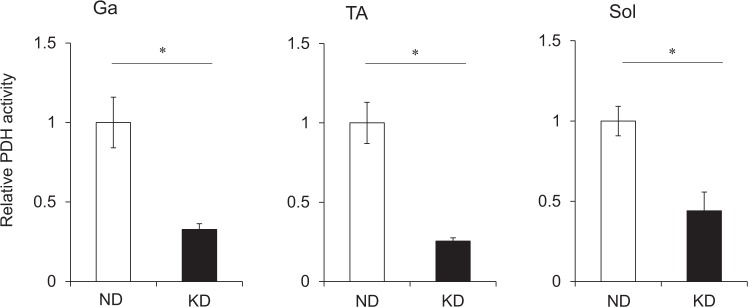
Pyruvate dehydrogenase activity in skeletal muscles. Activity of PDH in gastrocnemius (Ga), tibialis anterior (TA) and soleus (Sol) muscles of mice fed with normal (ND; unfilled squares) or ketogenic (KD; filled squares) diets for seven days. Results are shown as means ± SEM (n = 6 per group). Value for mice fed with ND is expressed as 1.0. ^*^P < 0.01 (ND vs. KD). PDH, pyruvate dehydrogenase.

**Figure 4:**
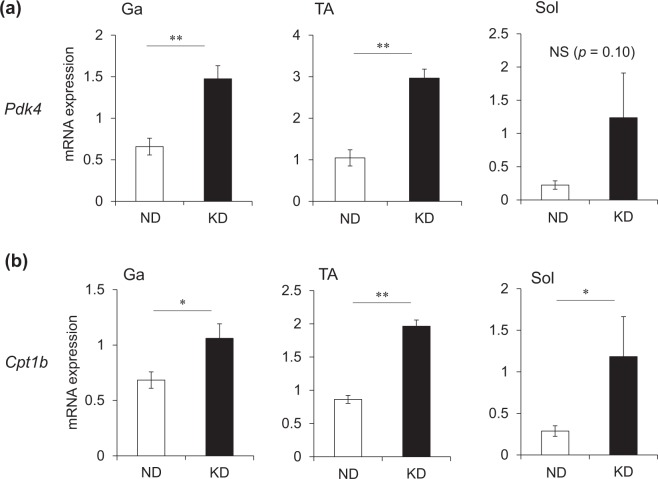
Messenger RNA expression of genes associated with glucose and lipid metabolism in skeletal muscles. Expression of *Pdk4* (**a**) and *Cpt1b* (**b**) mRNA in gastrocnemius (Ga), tibialis anterior (TA) and soleus (Sol) muscles of mice fed with normal (ND; unfilled squares) or ketogenic (KD; filled squares) diets for seven days. Results are shown as means ± SEM (n = 7–8 per group). ^*^P < 0.05 and ^**^P < 0.01 (ND vs. KD).

We measured plasma metabolic parameters to characterize the effects of the KD on metabolism. Plasma albumin levels as an index of nutritional status (Fig. 2a) were lower and plasma glucose levels were 60% decreased in mice fed with the KD, compared with the ND (Fig. 2b). Insulin levels were remarkably decreased at 0.15 ng/mL in mice fed with the KD (Fig. 2c) and they became hypoinsulinemic compared with ND mice. Unlike the early phase of starvation^16^, plasma alanine levels were lower in mice fed with the KD than the ND (Fig. 2d). Meanwhile, plasma β-hydroxybutyrate (ketone body) levels were about 10-fold higher in mice fed with the KD, than the ND (Fig. 2e). Circulating levels of free fatty acids (FFA) increased to 700% in the mice fed with the KD compared with the ND (Fig. 2f). The KD increased plasma corticosterone levels 2.9-fold (Fig. 2g) and decreased plasma IGF-1 levels by 60% compared with the ND (Fig. 2h). We compared energy metabolism in the skeletal muscles of mice fed with the KD and the ND by measuring activities of pyruvate dehydrogenase (PDH), which catalyzes the oxidative decarboxylation of pyruvate into acetyl-CoA and links glycolysis to the citric acid cycle. The activity of PDH was significantly decreased in the Ga, TA and soleus (Sol) muscles of mice fed with the KD (Fig. 3). The expression of pyruvate dehydrogenase kinase 4 (*Pdk4*) that contributes to PDH phosphorylation and impaired glucose utilization, was also increased 2.2-, 2.8- and 3.8-fold in the Ga, TA and Sol muscles, respectively, of mice fed with the KD, compared with the ND (Fig. 4a). The mRNA expression of carnitine palmitoyltransferase 1B (*Cpt1b*) that is involved in the transport of long-chain fatty acyl-CoA from cytoplasm into mitochondria was slightly, but significantly upregulated by the KD in the Ga, TA and Sol muscles (Fig. 4b). These results indicated that the KD shifted the energy substrate from glucose to fat.

### Ketogenic diet decreases muscle weight, fiber area and grip strength

**Figure 5:**
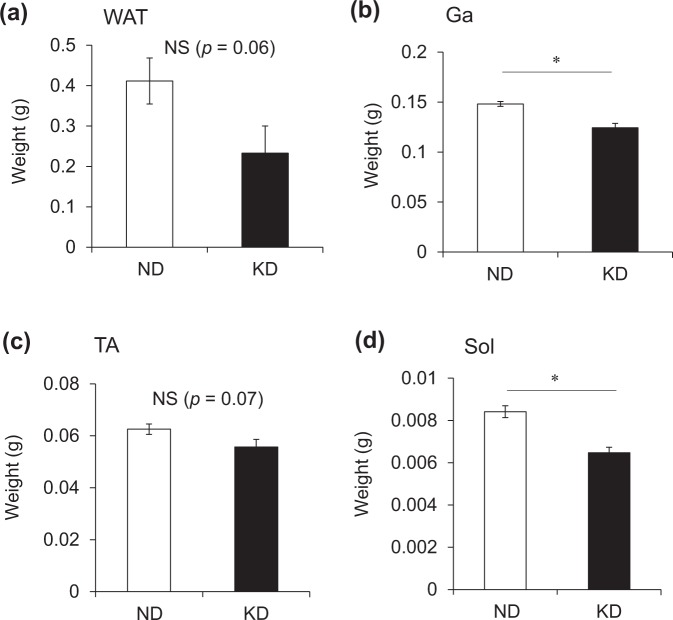
Weight of white adipose tissue and skeletal muscles. Weight of perigonadal white adipose tissue (WAT; **a**), gastrocnemius (Ga; **b**), tibialis anterior (TA; **c**), and soleus (Sol; **d**) muscles of mice fed with normal (ND; unfilled squares) or ketogenic (KD; filled squares) diets for seven days. Results are shown as means ± SEM (n = 7–8 per group). ^*^P < 0.01 (ND vs. KD).

**Figure 6:**
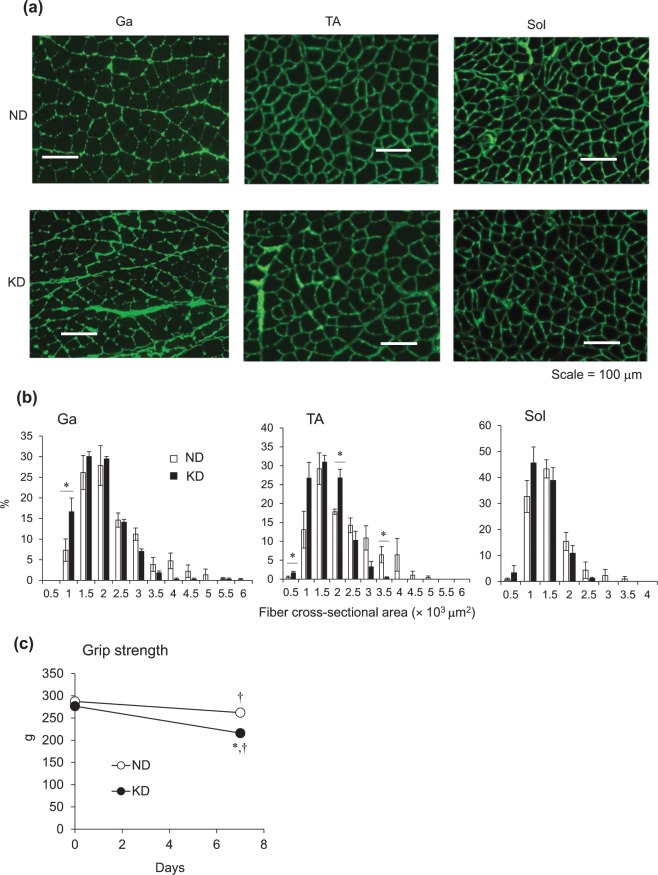
Measurements of muscle fiber area and grip strength. (**a**) Representative cross-sections of gastrocnemius (Ga), tibialis anterior (TA) and soleus (Sol) muscles from mice fed with normal (ND) or ketogenic (KD) diets for seven days. Scale bar = 100 μm. (**b**) Frequency histograms show distribution of cross-sectional areas of muscle fibers from mice fed with ND (unfilled squares) or KD (filled squares). Results are shown as means ± SEM (n = 3–4 per group). ^*^P < 0.01 (ND vs. KD). (**c**) Average grip strength in mice fed with normal (ND; unfilled circles) or ketogenic (KD; filled circles) diets for seven days. Results are shown as means ± SEM (n = 7–8 per group). ^*^P < 0.01 (ND vs. KD). ^†^*P* < 0.01 (Day 0 vs. 7).

We weighed the Ga, TA and Sol muscle tissues of mice fed with ND and KD. The KD tended to decrease fat mass (Fig. 5a), but the difference did not reach significance (*p* = 0.06), possibly because a large error was associated with this value. The wet weight of the Ga, TA and Sol muscles obviously decreased by 23%, 11% and 16%, respectively, in mice fed with the KD, compared with the ND, although the difference in TA weight between the groups did not reach significance (Fig. 5b–d). We then immunohistochemically analyzed cross-sections of Ga, TA and Sol muscles from mice fed with the ND and KD to determine whether the loss of muscle mass was reflected in a decrease in the size of individual fibers (Fig. 6a). The distribution of fibers in a histogram shifted leftwards and was smaller in mice fed with the KD (Fig. 6b). The distribution of those ranging from 500–1,000 and 3,000–6,000 μm^2^ was higher and lower, respectively, in mice fed with the KD. Muscle function evaluated by measuring grip strength did not significantly differ between the mice before starting the KD. Grip strength was 22% and 9% decreased in the mice fed with the KD and ND, respectively (Fig. 6c).

### Ketogenic diet upregulates muscle atrophy-related and glucocorticoid receptor target genes

**Figure 7:**
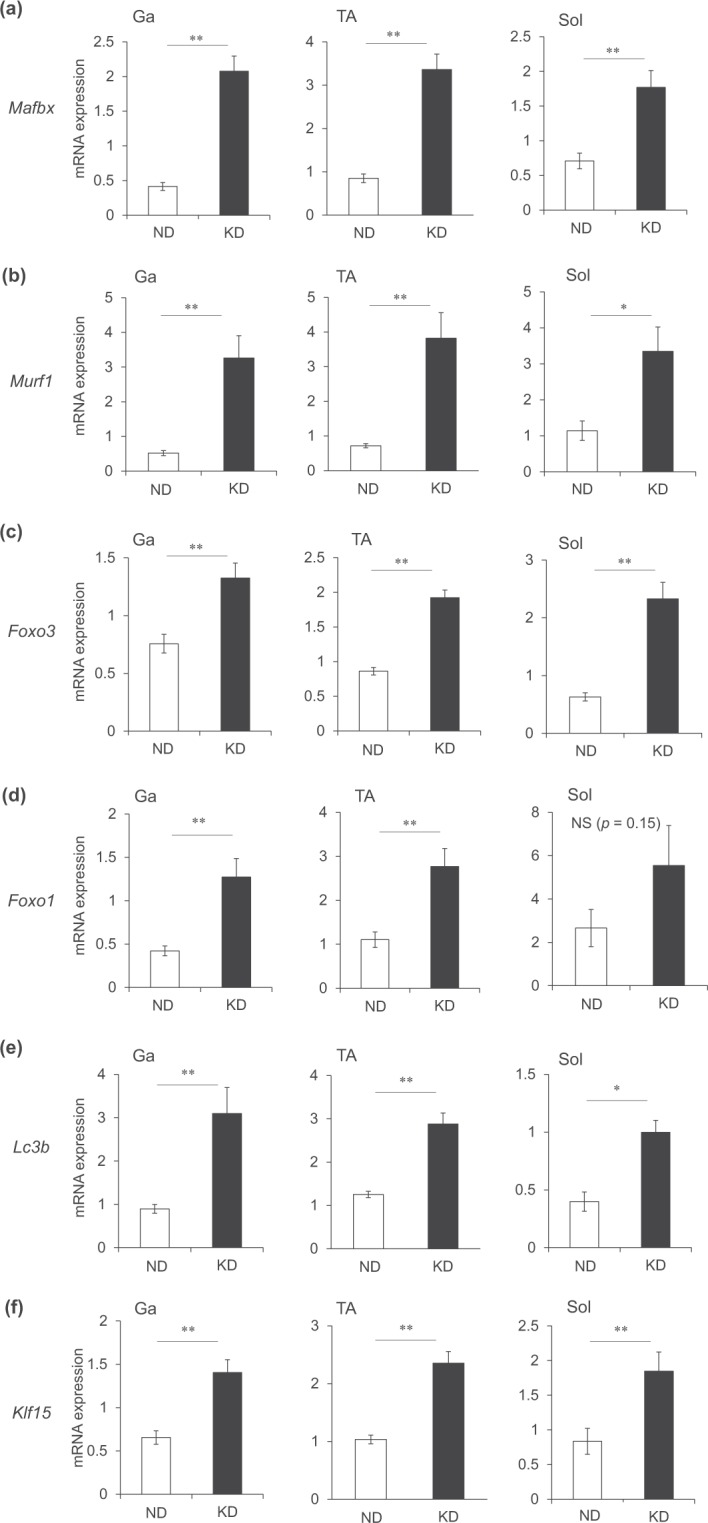
Messenger RNA expression of genes associated with muscle atrophy in skeletal muscles. Expression of genes associated with muscle atrophy and glucocorticoid receptor targets in gastrocnemius (Ga), tibialis anterior (TA) and soleus (Sol) muscles of mice fed with normal (ND; unfilled squares) or ketogenic (KD; filled squares) diets for seven days. Results are shown as means ± SEM (n = 7–8 per group). ^*^P < 0.05 and ^**^P < 0.01 (ND vs. KD).

**Figure 8:**
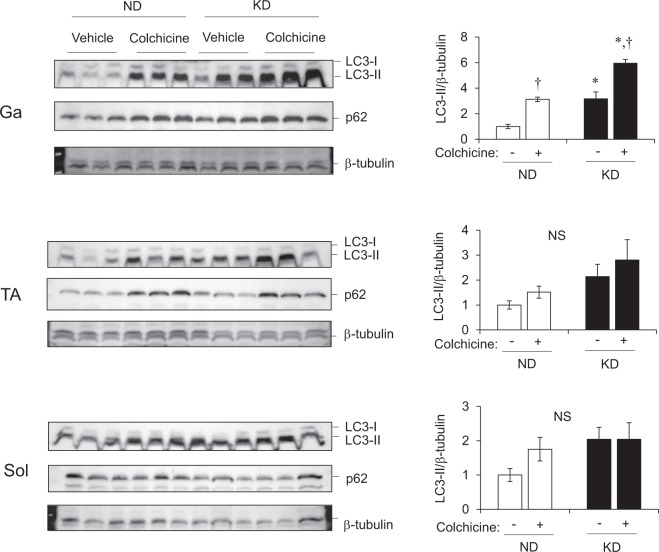
Protein expression of molecules associated with autophagy in skeletal muscle. Protein expression of LC3 and p62. Mice were fed with normal (ND) or ketogenic (KD) diets for seven days, then intraperitoneally administered with colchicine 4 h before collecting gastrocnemius (Ga), tibialis anterior (TA) and soleus (Sol) muscles. Band intensity is expressed as ratios of LC3-II to β-tubulin quantified by densitometry. Results are shown as means ± SEM (n = 3 per group). ^*^P < 0.01 (ND vs. KD). ^†^*P* < 0.01 (vehicle vs. colchicine). Supplemental Fig. 2 shows full-length gels.

We investigated how the loss of muscle weight under a KD correlates with gene expression in the Ga, TA and Sol muscles. The expression of *Mafbx* and *Murf1* that encode muscle atrophy-related ubiquitin ligases in mice fed with KD and ND, was 5.0-, 4.0- and 2.5-fold and 6.3-, 5.3- and 2.9-fold higher in the Ga, TA and Sol muscles, respectively (Fig. 7a,b). The transcription factors for these genes, *Foxo3* and *Foxo1*, were also upregulated by the KD in all muscles we evaluated (Fig. 7c,d). Transcriptional activation of the autophagy gene, *Lc3b*, was upregulated 3.5-, 2.3- and 2.5-fold in the Ga, TA and Sol muscles, respectively, by the KD compared with the ND (Fig. 7e). The direct GR target gene^13^, *Klf15*, was also upregulated about 2-fold in the Ga, TA and Sol muscles of mice fed with the KD compared with the ND (Fig. 7f). We compared autophagy flux *in vivo* in mice under ND and KD. Colchicine increased the amount of LC3-II in all three muscles in the ND group. The KD increased LC3-II values and KD plus colchicine increased them above that observed with KD alone or with ND plus colchicine in Ga and TA muscles (Fig. 8). The KD alone increased LC3-II values in Sol muscles, but adding colchicine did not increase the intensity of LC3-II band. We found that colchicine increased p62 in the Ga and TA, but not in the Sol muscles of both groups of mice. These results indicated that the KD enhanced autophagy, particularly in the Ga muscles (Fig. 8).

### Ketogenic diet upregulates oxidative stress-responsive genes

**Figure 9:**
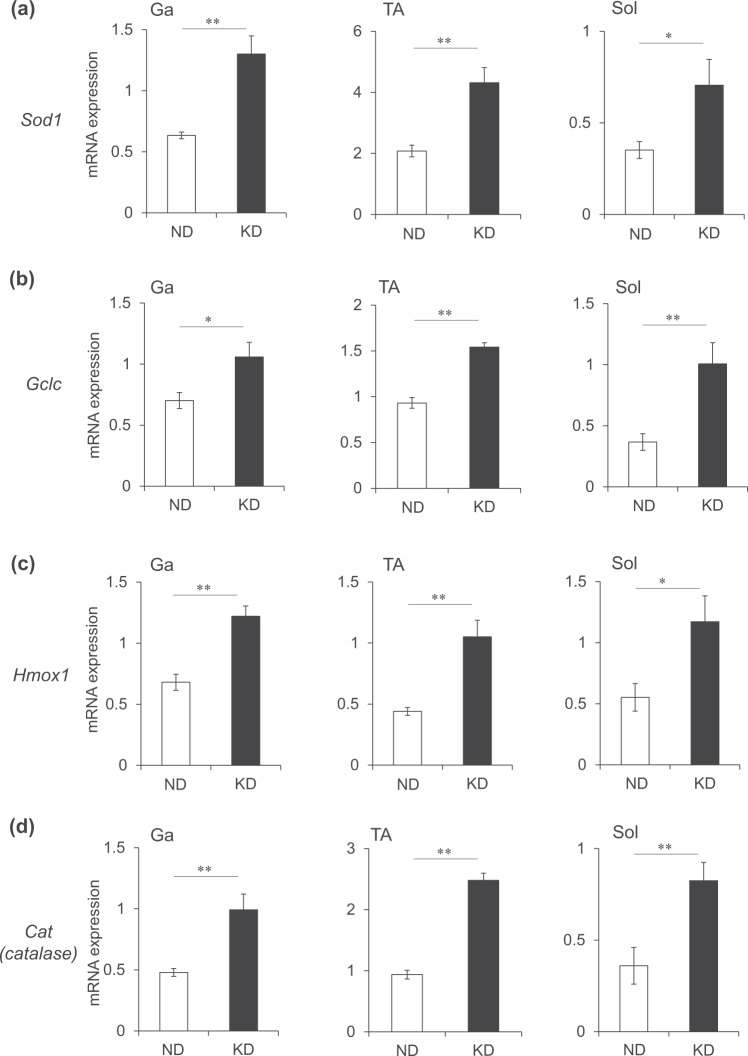
Messenger RNA expression of oxidative stress-responsive gene in skeletal muscles. Expression of oxidative stress-responsive genes in the gastrocnemius (Ga), tibialis anterior (TA) and soleus (Sol) muscles of mice fed normal (ND; unfilled squares) or ketogenic (KD; filled squares) diets for seven days. Results are shown as means ± SEM (n = 7–8 per group). ^*^P < 0.05 and ^**^P < 0.01 (ND vs. KD).

A high-fat diet increases the production of reactive oxygen species (ROS)^17^ and the expression of genes associated with muscle atrophy^18,19^. We examined the expression of the antioxidant genes, superoxide dismutase 1 (*Sod1*), glutamate-cysteine ligase catalytic subunit (*Gclc*), heme oxygenase 1 (*Hmox1*) and catalase (*Cat*) that are upregulated in response to oxidative stress. All of these antioxidant genes were upregulated 2-fold in the Ga, TA and Sol muscles in mice fed with the KD compared with the ND (Fig. 9).

### Ketogenic diet downregulates genes associated with muscle anabolism

**Figure 10:**
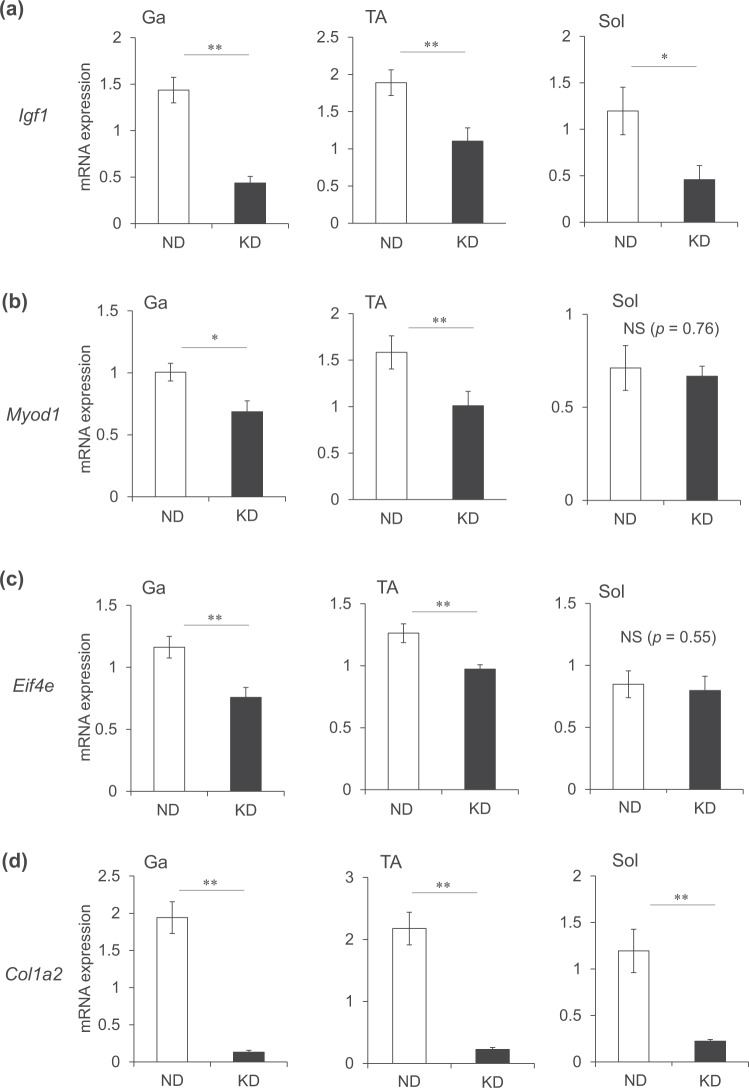
Messenger RNA expression of muscle anabolism-related gene in skeletal muscles. Expression of muscle growth-related genes in gastrocnemius (Ga), tibialis anterior (TA) and soleus (Sol) muscles of mice fed with normal (ND; unfilled squares) or ketogenic (KD; filled squares) diets for seven days. Results are shown as means ± SEM (n = 7–8 per group). ^*^P < 0.05 and ^**^P < 0.01 (ND vs. KD).

**Figure 11:**
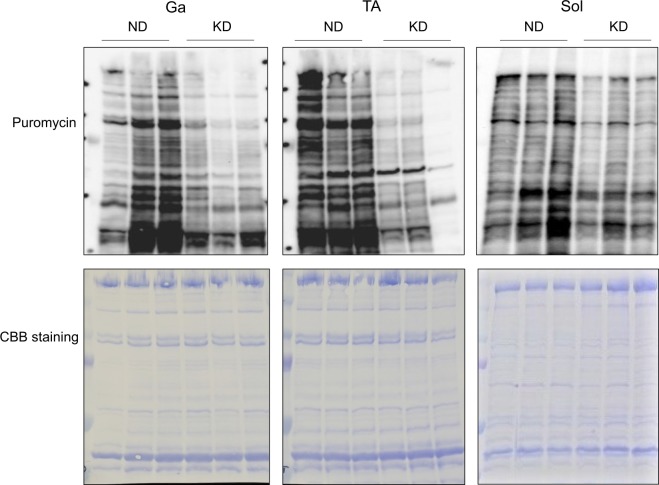
Measurement of protein synthesis by SUnSET method. Mice were fed with normal (ND) or ketogenic (KD) diets for seven days, then intraperitoneally administered with puromycin 30 min before collecting gastrocnemius (Ga) tibialis anterior (TA) and soleus (Sol) muscles to detect peptides labeled with puromycin by immunoblotting.

We examined the mRNA expression of genes associated with muscle anabolism. Insulin-like growth factor-1 plays a key role in muscle hypertrophy^20^. In accordance with plasma IGF-1 values (Fig. 2h), the mRNA expression of *Igf1* was decreased by 30% in the Ga and Sol muscles, and by 58% in the TA muscles of mice fed with the KD (Fig. 10a). Myogenic differentiation 1 (MyoD1) is a major transcription factor that mediates the transactivation of myofibrillar genes such as myosin heavy chain and skeletal α-actin^21–23^. The mRNA expression of *Myod1* was significantly decreased in the Ga and TA muscles of mice fed with the KD, but essentially identical in the Sol muscles of mice fed with the KD and the ND (Fig. 10b). The KD also decreased the mRNA expression of eukaryotic translation initiation factor 4E (*Eif4e*) that is involved in the initiation of protein synthesis in the Ga and TA, but not in the Sol (Fig. 10c). The KD decreased the expression of the gene encoding collagen that is an extracellular matrix protein in all muscles examined (Fig. 10d). These results indicated that the synthesis of intracellular and extracellular matrix proteins was generally reduced in the skeletal muscles of mice fed with a KD, and this also occurs in fasting rodents^24^. Furthermore, surface sensing of translation (SUnSET) analysis using puromycin confirmed that the KD remarkably decreased the amounts of puromycin-labeled peptides, indicating that the KD suppressed muscle protein synthesis in mice (Fig. 11).

## Discussion

**Figure 12:**
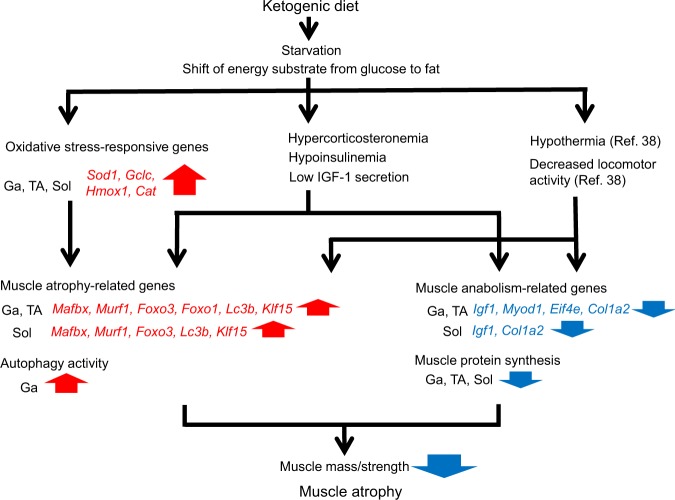
Overview of ketogenic diet effects on skeletal muscle. *Cat*, catalase; *Col1a2*, collagen type I alpha 2; *Eif4e*, eukaryotic translation initiation factor 4E; *Foxo*, forkhead box O; *Klf15*, kruppel-like factor 15; Ga, gastrocnemius muscle; *Gclc*, glutamate-cysteine ligase catalytic subunit; *Hmox1*, heme oxygenase 1; *Igf1*, insulin-like growth factor 1; *Lc3b* (*Map1lc3b*), microtubule-associated protein 1 light chain 3 beta; *Mafbx*, muscle atrophy F-box; *Murf1*, muscle RING finger 1; *Myod1*, myogenic differentiation 1; *Sod1*, superoxide dismutase 1; Sol; soleus muscle; TA, tibialis anterior muscle.

Chronically poor nutritional status accompanied by anorexia is considered to be a factor that causes muscle atrophy under pathological conditions such as sarcopenia, chronic obstructive pulmonary disease (COPD), cancer cachexia, and anorexia nervosa^25,26^. Food has been withdrawn from experimental animal models in efforts to elucidate the molecular basis of short-term starvation. However, these studies were more focused on acute and transient responses of skeletal muscles. Here, we created a new experimental model of starvation-induced muscle wasting, by feeding mice with a KD. This model allowed evaluation of the chronic effects of starvation on muscle atrophy, unlike the conventional model in which food is withdrawn for only 24–48 h to ensure the welfare of rodents^24^. Consuming a KD decreases circulating levels of albumin, glucose and insulin, and increases ketone bodies and FFA, indicating that consuming a KD modeled persistent starvation in which the primary fuel source shifts from glucose to ketones generated by FFA oxidation. The KD caused the loss of muscle mass accompanied by decreased plasma insulin and IGF-1 and increased plasma corticosterone values, then upregulated GR-target ubiquitin ligases and autophagy marker genes in the Ga, TA and Sol muscles. Hu *et al*. reported that the stimulation of muscle proteolysis requires increased endogenous GC and impaired insulin signaling^27^. The attenuation of IGF-1 signaling also accelerates muscle atrophy^20^. *In vivo* autophagy flux assay revealed that KD mainly activated autophagy in the Ga muscle. Based on these findings, we concluded that hypercorticosteronemia and hypoinsulinemia, along with decreased IGF-1 secretion induced by the KD, resulted in muscle atrophy via autophagy, particularly in the Ga muscle. On the other hand, expression of the antioxidant genes *Sod1*, *Gclc*, *Hmox1* and *Cat* was upregulated in the Ga, TA and Sol muscles, suggesting that the KD caused ROS generation; nonetheless, the antioxidant system in skeletal muscles remained relatively intact. Although the molecular mechanism remains unknown, the KD downregulated mRNA expression of the muscle anabolism-related genes, *Igf1* and *Col1a2*, indicating a decline in muscle anabolism. SUnSET analyses confirmed that the KD suppressed muscle protein synthesis in all muscles evaluated. These finding suggest that suppressed protein synthesis contributed to KD-induced muscle atrophy in the TA and Sol, whereas accelerated autophagy and reduced protein synthesis synergistically induced atrophy in the Ga muscle. Along with the loss of muscle mass, immunohistochemical findings revealed smaller myofibers in the Ga, TA and Sol muscles of KD mice. We concluded that feeding rodents with a KD is a useful and practical way to investigate the effects of chronic starvation on skeletal muscle (Fig. 12).

In addition to hypercorticosteronemia, we postulated that oxidative stress contributes to muscle atrophy in mice fed with a KD. The expression of genes encoding antioxidant enzymes was increased by the KD, although whether a KD causes oxidative stress remains controversial^28^. We measured 4-hydroxy-2-nonenal (HNE)-conjugated cytosolic protein as an indicator of lipid peroxidation and found that 4-HNE levels were essentially identical between ND and KD mice (Supplementary Fig. 1). An activated antioxidant system could improve the mitochondrial redox state after consuming a KD. Further studies are needed to confirm the involvement of ROS production on muscle atrophy caused by a KD.

The weight of the TA did not significantly differ between mice fed with the ND or the KD, although gene expression was similar among all muscles evaluated. We found that hypercorticosteronemia contributes to the induction of muscle atrophy while on a KD. The TA muscles comprise fast twitch-muscle fibers that are sensitive to GC-induced muscle wasting. We speculate that an unidentified mechanism contributes to the maintenance of TA weight, although this awaits confirmation.

The KD increased levels of the circulating ketone, β-hydroxybutyrate. This metabolic substrate works not only to produce energy during prolonged fasting, but also as a signaling molecule. It is an endogenous and specific inhibitor of class I histone deacetylases (HDACs), it promotes the hyperacetylation of histone proteins and contributes to an open chromatin environment that leads to induction of the transcription factor FOXO3^29^. Activated FOXO might accelerate the upregulation of *Mafbx* and *Murf1* mRNA during KD consumption.

Calorie intake could not account for the decrease in BW on day 3. Depleted hepatic and muscle glycogen stores along with associated bound water are thought to decrease BW during the initial phase of extreme carbohydrate restriction^15^. We postulated that the depletion of glycogen stores decreased the amount of water stored with glycogen, resulting in increased water excretion and a loss of BW at the initial phase of KD consumption in our model mice.

The KD reduced plasma IGF-1 levels in the present study. A growth hormone (GH)-GH receptor cascade positively regulates *Igf1* mRNA transcription^30^. Reports have indicated that GH values are normal^31^ or elevated^32^, whereas circulating IGF-1 levels are reduced in rodents fed with a KD^31^. Our findings were similar to these. The KD might have caused GH resistance^33^ which could have been responsible for the IGF-1 reduction. Further studies are required to elucidate the underlying mechanism of the IGF-1 reduction in mice fed with a KD.

A KD is of interest to humans because it has been applied as a weight-loss strategy^15^ and to treat epilepsy^34^. However, skeletal muscle physiology might be at risk due to hypercorticosteronemia, hypoinsulinemia, decreased IGF-1 secretion and an oxidized redox environment associated with chronic KD consumption. However, little is known about the adverse effects of a KD on skeletal muscle.

Alanine released from skeletal muscle during starvation in response to increased protein degradation for hepatic gluconeogenesis results in a decrease in muscle mass^16^. Dyar *et al*. identified increased serum alanine values in mice fed with a high-fat diet for 10 weeks, possibly via muscle proteolysis^35^. However, they noted that serum creatinine was increased on the high-fat diet, indicating that muscle mass was maintained and that rates of protein turnover increased^35^. The present study found lower plasma alanine levels in mice fed with the KD, compared with the ND, suggesting the slow release of amino acids from skeletal muscle or accelerated hepatic gluconeogenesis and alanine consumption. Plasma alanine levels are lower in patients with chronically poor nutrition, such as those with COPD^36^, as well as in aged mice^37^, although the mechanism remains obscure. A KD might replicate the slower protein degradation associated with chronically poor nutrition. When food is unavailable, experimental animals become hyperactive for food. Mice become hyperactive after one or two days on a KD, whereas chronic continuous KD consumption gradually decreases locomotor activity and body temperature^38^. Based on these facts, physiological adaptation to a KD might somewhat resemble the chronically poor nutritional status associated with aging and COPD (Table 1). Although poor nutritional status shares some features with acute forms of muscle atrophy caused by food withdrawal, sciatic denervation and synthetic GC administration, food withdrawal from experimental animals is of limited value to understanding the effects of chronic poor nutritional status on skeletal muscle. Feeding experimental animals with a KD is a novel model of starvation, which might more closely resemble the chronically poor nutrition associated with conditions such as COPD and anorexia, as well as aging.

**Table 1:** Comparison of ketogenic diet consumption with other animal models of skeletal muscle atrophy.

	Ketogenic diet	Food deprivation	High-fat diet	Sciatic denervation	Aging
Plasma glucocorticoid	Increase	Increase^42^	Unchanged^35^	Unchanged^43^	Slight increase^44^
Insulin	Decrease	Decrease^45^	Increase^46^	Unchanged (Insulin binding to muscle)^47^	Increase^48^
Alanine release from skeletal muscle	Decrease	Increase^45^	Increase^35^	Increase^49^	Decrease^37^
Muscle atrophy-related gene expression	Increase	Increase^24^	Unchanged^46^	Increase^6^	Unchanged^50^
Physical activity	Decrease^38^	Increase^51^	Unchanged^52,53^	Unchanged^54^	Decrease^37^
Body temperature	Decrease^38^	Decrease^55^	Nighttime decrease; daytime increase^53^	Unchanged^43,54^	Decrease^56^

## Methods

### Animal care and handling

All animal experiments proceeded according to the guidelines for animal experiments at the National Institute of Advanced Industrial Science and Technology (AIST). The Animal Care and Use Committees at AIST approved all the experimental protocols described herein (Permission #166).

Six-week-old female Jcl:ICR mice (Japan SLC Inc., Shizuoka, Japan) were housed with access to a standard diet (AIN-93M: Oriental Yeast Co. Ltd., Tokyo, Japan) and water *ad libitum* for four weeks under 12 h light–12 h dark cycles. The mice were fed with either the AIN-93M (Oriental Yeast Co. Ltd., Tokyo, Japan) normal diet (ND) or the modified AIN-93 ketogenic (KD) diet (73.9% fat, 8.3% protein and 0.73% carbohydrate, w/w; Oriental Yeast Co. Ltd.) for seven days. The caloric contents of the ND and KD were 389 and 702 kcal/100 g diet, respectively The proportions of calories derived from fat, carbohydrate and protein were 10.3%, 75.4% and 14.2%, respectively, in the ND and 94.8%, 0.1% and 4.8%, respectively, in the KD. The mice were sacrificed, then the Ga, TA and Sol, as well as perigonadal white adipose tissues (WAT) were dissected, weighed and frozen in liquid nitrogen.

### Real-time reverse transcription-polymerase chain reaction (RT-PCR)

Total RNA was extracted using guanidinium thiocyanate followed by RNAiso Plus (Takara Bio Inc., Otsu, Japan). Single-stranded cDNA was synthesized using PrimeScript™ RT reagent kits with gDNA Eraser (Takara Bio). Real-time RT-PCR proceeded using SYBR^®^ Premix Ex Taq™ II (Takara Bio) and a LightCycler™ (Roche Diagnostics, Mannheim, Germany). The amplification conditions comprised 95 °C for 10 s followed by 45 cycles of 95 °C for 5 s, 57 °C for 10 s and 72 °C for 10 s. Supplemental Table 1 shows the primer sequences. Amounts of target mRNA were normalized relative to that of *Actb*.

### Measurement of plasma metabolic parameters

Blood collected in EDTA-coated tubes was immediately separated by centrifugation for 10 min at 5,800 × *g*. Plasma was collected and stored at −80 °C. Plasma albumin, glucose, insulin, alanine, β-hydroxybutyrate, free fatty acid (FFA), glucocorticoid, and IGF-1 values were measured using A/G B-Test Wako (Wako Pure Chemical Industries, Osaka, Japan), Glucose CII-test Wako (Wako), Mouse Insulin ELISA kits (Morinaga Institute of Biological Science, Kanagawa, Japan), Alanine Colorimetric/Fluorometric Assay Kits (BioVision, Milpitas, CA, USA), β-Hydroxybutyrate (β-HB) Colorimetric Assay Kits (BioVision), NEFA C-test Wako (Wako), AssayMax corticosterone ELISA kits (AssayPro, St. Charles, MO, USA) and Mouse/Rat IGF-1 Quantikine ELISA kit (R&D Systems, Inc., Minneapolis, MN, USA) respectively.

### PDH activity

The skeletal muscles were frozen and homogenized in liquid nitrogen, lysed with ice cold PDH assay buffer (BioVision), and separated by centrifugation for 5 min at 10,000 × *g*. The protein content in the supernatant was measured using Protein Assay BCA Kits (Wako). The activity of PDH was determined in 300 μg of protein lysate using Pyruvate Dehydrogenase Activity Colorimetric Assay Kit (BioVision).

### Immunohistochemistry

Muscle cryosections were incubated with 5% Block Ace Powder (DS Pharma Biomedical Co. Ltd., Osaka, Japan) in PBS, and incubated with 1:500-diluted anti-laminin antibody (SIGMA, St. Louis, MO, USA). The specimens were washed with PBS and incubated with secondary antibodies labeled with 1:500-diluted Alexa Fluor 488 (Thermo Fisher Scientific, Waltham, MA, USA). Immunostained specimens were mounted in Dako Fluorescent Mounting Medium (Agilent Technologies, Santa Clara, CA, USA). The size of muscle fibers was measured using a Biozero BZ-9000 fluorescence microscope (KEYENCE, Osaka, Japan).

### *In vivo* autophagy flux assay

The activity of autophagy was measured as described^39^. The mice were injected intraperitoneally (i.p.) with 0.4 mg/kg BW of colchicine (SIGMA; dissolved in saline) at 4 h before collecting muscle samples. Control mice received an equal volume of saline i.p.

### Puromycin injection

We measured protein synthesis using surface sensing of translation (SUnSET) method^40,41^. The mice were injected i.p. with 0.04 μmol/g BW of puromycin (Wako; dissolved in PBS) at 30 min before collecting muscle samples. Amounts of puromycin incorporated into total protein were determined by immunoblotting as described below.

### Immunoblotting

Proteins were extracted from mouse muscles into RIPA buffer (Wako) supplemented with protease and phosphatase inhibitor tablets (Roche Diagnostics) and 10 μM MG132 (Cayman Chemical, Ann Arbor, MI, USA). Total protein extracts were resolved by SDS-polyacrylamide gel electrophoresis and transferred to polyvinylidene difluoride membranes. Nonspecific binding on membranes was blocked with 4% Block Ace Powder (DS Pharma Biomedical Co. Ltd.) and then the membranes were incubated with primary antibodies at 4 °C overnight. Bound antibodies were detected by chemiluminescence by using Immuno Star LD (Wako) and signals were quantified by densitometry. The primary antibodies were anti-LC3 (SIGMA), anti-puromycin (Merck Millipore, Burlington, MA, USA) and anti-β-tubulin (SIGMA).

### Statistical analysis

Values are expressed as means ± SEM and were statistically evaluated by Student *t*-tests using Excel-Toukei 2015 software (Social Survey Research Information Co. Ltd., Osaka, Japan). Data about BW, calorie intake, grip strength and LC3 band intensity were statistically evaluated using two-way analyses of variance (ANOVA; Supplemental Table 2) and Tukey-Kramer multiple comparison tests using Excel-Toukei 2015 software. Differences were considered significant at *P* < 0.05.

## Supplementary information

Supplementary information
