# Supplementary material for: Ketogenic diet induces skeletal muscle atrophy via reducing muscle protein synthesis and possibly activating proteolysis in mice
Source: Sci Rep. 2019 Dec 23;9:19652. doi: 10.1038/s41598-019-56166-8 (PMC6928149; doi:10.1038/s41598-019-56166-8)
Supplement: Supplementary file 1 — Supplementary information [file 41598_2019_56166_MOESM1_ESM.pdf]

Ketogenic diet induces skeletal muscle atrophy via reducing muscle protein synthesis and possibly activating proteolysis in mice

Reiko Nakao[1*], Tomoki Abe[1*], Saori Yamamoto[1], Katsutaka Oishi[1,2,3,4]

[1]Biological Clock Research Group, Biomedical Research Institute, National Institute of Advanced Industrial Science and Technology (AIST), Tsukuba, Ibaraki 305-8566, Japan

[2]Department of Applied Biological Science, Graduate School of Science and Technology, Tokyo University of Science, Noda, Chiba 278-8510, Japan

[3]Department of Computational and Medical Sciences, Graduate School of Frontier Sciences, The University of Tokyo, Kashiwa, Chiba 277-0882, Japan

[4]School of Integrative and Global Majors (SIGMA), University of Tsukuba, Tsukuba, Ibaraki 305-8577, Japan

[*] These authors contributed equally to this work.

Supplementary information

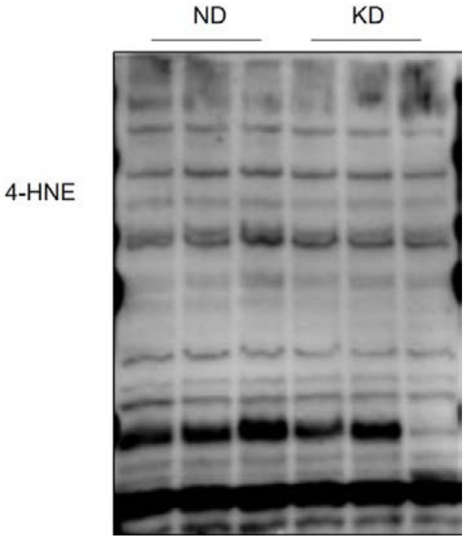

Supplemental Figure 1. Lipid peroxide did not accumulate under a ketogenic diet. Levels of 4-hydroxy-2-nonenal (HNE)-conjugated cytosolic protein (indicator of lipid peroxidation) in gastrocnemius muscles of mice fed with normal (ND) or ketogenic (KD) diets for seven days. Proteins extracted from muscle were immunoblotted using anti-4HNE (Japanese Institute for the Control of Aging, Fukuroi, Japan).

Ga

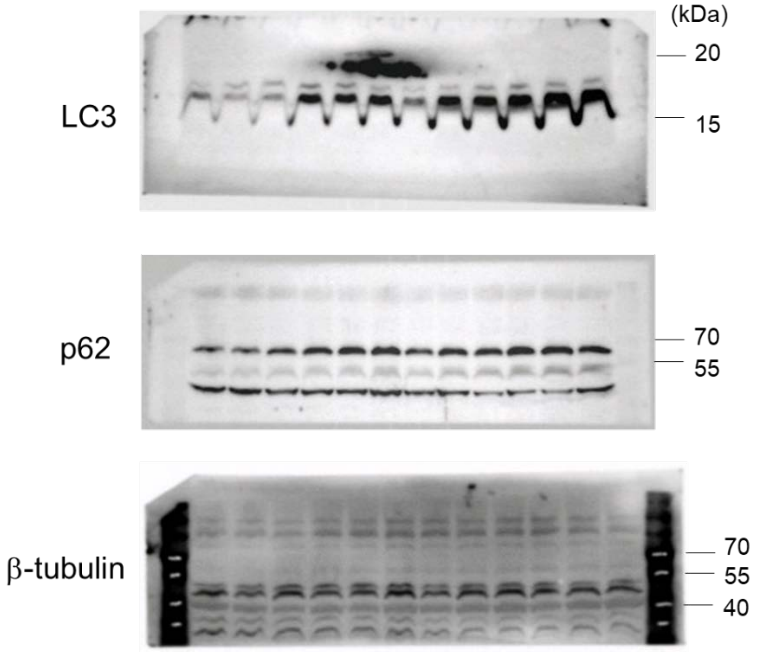
(kDa)
LC3
20
15
p62
70
55
β-tubulin
70
55
40

TA

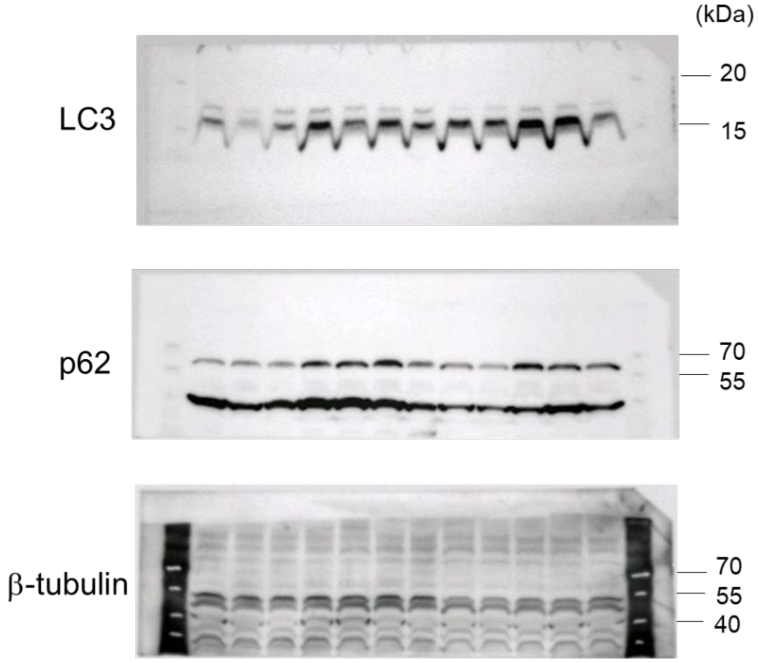
(kDa)
LC3
20
15
p62
70
55
β-tubulin
70
55
40

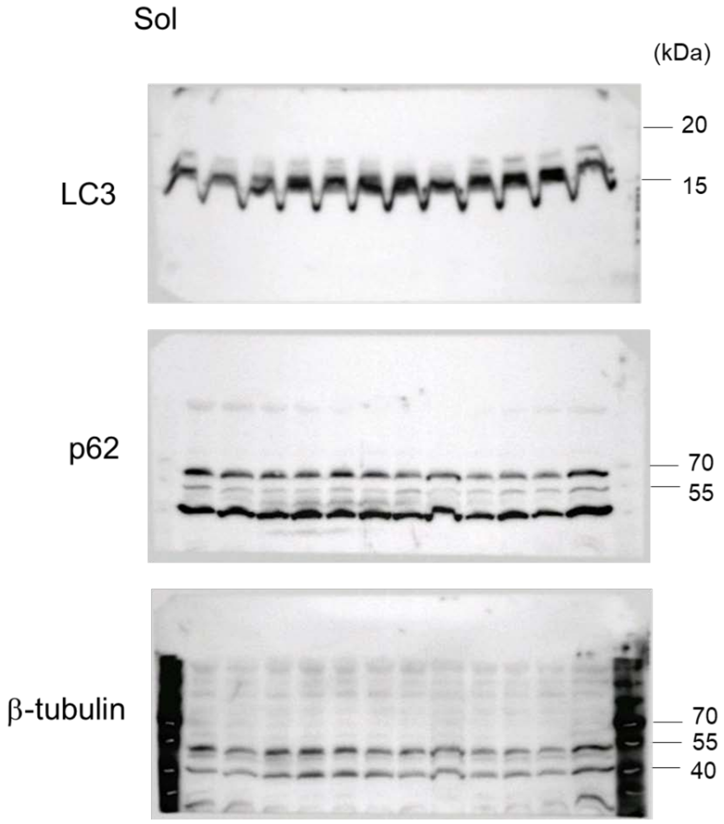

Supplemental Figure 2. Full-size blots on polyacrylamide gels.

Actual images of gel blots of LC3, p62 and β-tubulin shown in Figure 8. Images vary in size because membrane was cut to blot several proteins.

Supplemental Table 1. Primer sequences for real-time RT-PCR.

Gene	Forward primer sequence (5’ to 3’)	Reverse primer sequence (5’ to 3’)
Cpt1b	TGAGACACATCTACCTGTCTGG	CCATGGAGATGTCCACCTTGC
Pdk4	CACATGCTCTTCGAACTCTTCAAG	TGATTGTAAGGTCTTCTTTTCCCAAG
Mafbx	GGAAGCTTTCAACAGACTGGA	CTCAGGGATGTGAGCTGTGA
Murf1	ACGAGAAGAAGAGCGAGCTG	CTTGGCACTTGAGAGGAAGG
Foxo3	ACGAGTGGATGGTGCGCTGTGTGC	TCATTCTGAACGCGCATGAAGCG
Foxo1	TCACCAAGGCCATCGAGAGCTC	CCTCTGGATTGAGCATCCACCAAG
Lc3b	CGTCCTGGACAAGACCAAGT	ATTGCTGTCCCGAATGTCTC
Klf15	CCAGGCTGCAGCAAGATGTACAC	TGCCTTGACAACTCATCTGAGCGG
Sod1	GGAAGCATGGCGATGAAA	AAATGAGGTCCTGCACTGGTA
Gclc	ATTGTTATGGCTTTGAGTGCTGC	CCTCCCGTGTTCTATCATCTACAG
Hmox1	CGCCTTCCTGCTCAACATT	TGTGTTCCTCTGTCAGCATCAC
Cat	GGTAACTGGGATCTTGTGGGAAAC	CTGTGGGTTTCTCTTCTGGCTATG
Igf1	TGGATGCTCTTCAGTTCGTG	GTCTTGGGCATGTCAGTGTG
Myod1	TGAGCAAAGTTAATGAGGCCTTCG	TGCAGACCTTCGATGTAGCGGAT
Eif4e1	AGGACGGTGGCTGATCACA	TCTCTAGCCAGAAGCGATCGA
Col1a2	CAGAACATCACCTACCACTGCAA	TTCAACATCGTTGGAACCCTG
Actb	CACACCTTCTACAATGAGCTGC	CATGATCTGGGTCACTTTTCA

Supplemental Table 2. Two-way ANOVA of body weight, calorie intake, grip strength, and LC3 band intensity in mice fed with ketogenic diet (KD) or normal diet.

Factor		*F*	*P*
Body weight decrease	KD	58.40	< 0.001
	Days	81.69	< 0.001
	KD × days	40.44	< 0.001
Calorie intake	KD	3.21	0.089
	Days	13.89	< 0.001
	KD × days	9.72	< 0.001
Grip strength	KD	21.77	< 0.001
	Days	31.93	< 0.001
	KD × days	5.46	0.034
LC3 band intensity in Ga muscle	KD	56.30	< 0.001
	Colchicine	54.72	< 0.001
	KD × colchicine	0.98	0.35
LC3 band intensity in TA muscle	KD	5.94	0.041
	Colchicine	1.40	0.27

	KD × colchicine	0.022	0.89
LC3 band intensity in Sol muscle	KD	3.45	0.10
	Colchicine	1.11	0.32
	KD × colchicine	1.11	0.32

Ga, gastrocnemius; Sol, soleus; TA, tibialis anterior.
